# Supplementary material for: Optimising an Infusion Protocol Containing Cefepime to Limit Particulate Load to Newborns in a Neonatal Intensive Care Unit
Source: Pharmaceutics. 2021 Mar 8;13(3):351. doi: 10.3390/pharmaceutics13030351 (PMC8001063; doi:10.3390/pharmaceutics13030351)
Supplement: Supplementary file 1 [file pharmaceutics-13-00351-s001.pdf]

pharmaceutics

MDPI

Supplementary Materials: Optimising an Infusion Protocol Containing Cefepime to Limit Particulate Load to Newborns in a Neonatal Intensive Care Unit

Anthony Martin Mena, Morgane Masse, Laura Négrier, Thu Huong Nguyen, Bruno Ladam, Laurent Storme, Christine Barthélémy, Pascal Odou, Stéphanie Genay and Bertrand Décaudin

Table S1. Number of particles ≥2 µm, ≥5 µm, ≥10 µm, ≥15 µm, ≥25 µm and total for both infusion sets over the period T0-T8H. Results are expressed as medians [minimum; maximum] (Mann-Whitney test, $p < 0.05$, $n = 6$).

Particles T0-T8H	CEF Infusion Set	CAF Infusion Set	P
**≥2 µm**	56,465 [22,352–68,051]	2391 [990–3145]	0.0022
**≥5 µm**	9794 [3087–17,064]	383 [227–852]	0.0022
**≥10 µm**	1238 [338–1884]	127 [14–189]	0.0022
**≥15 µm**	342 [83–526]	43 [1–58]	0.0022
**≥25 µm**	59 [14–64]	3 [0–33]	0.0043
**Total**	89,389 [44,763–104,972]	4982 [1609–6159]	0.0022

Table S2. Number of particles ≥2 µm, ≥5 µm, ≥10 µm, ≥15 µm, ≥15 µm, ≥25 µm and total for both infusion sets over the period T4H-T5H. Results are expressed as medians [minimum; maximum] (Mann-Whitney test, $p < 0.05$, $n = 6$).

Particles T4H-T5H	CEF Infusion Set	CAF Infusion Set	P
**≥2 µm**	55,141 [19,641–66,060]	817 [215–2230]	0.0022
**≥5 µm**	9475 [2028–16,688]	136 [58–665]	0.0022
**≥10 µm**	1168 [327–1837]	19 [2–189]	0.0022
**≥15 µm**	331 [81–516]	6 [0–50]	0.0022
**≥25 µm**	58 [14–64]	1 [0–4]	0.0022
**Total**	86,246 [40,160–100,874]	1760 [376–4544]	0.0022

Table S3. Number of particles ≥2 µm, ≥5 µm, ≥10 µm, ≥15 µm, ≥25 µm and total for both infusion sets over the period T0-T8H. Results are expressed as medians [minimum; maximum] (Mann-Whitney test, $p < 0.05$, $n = 6$).

Particles T0-T8H	CEF Infusion Set with Vancomycin	CEF Infusion Set without Vancomycin	P
**≥2 µm**	56,465 [22,352–68,051]	55,710 [38,181–88,563]	0.9372
**≥5 µm**	9794 [3087–17,064]	13,874 [10,237–23,064]	0.0931
**≥10 µm**	1238 [338–1 884]	1426 [1305–2274]	0.0649
**≥15 µm**	342 [83–526]	552 [349–938]	0.0649
**≥25 µm**	59 [14–64]	74 [27–249]	0.5887
**Total**	89,389 [44,763–104,972]	80,315 [60,218–125,714]	0.6991

Table S4. Number of particles ≥2 µm, ≥5 µm, ≥10 µm, ≥15 µm, ≥25 µm and total for both infusion sets over the period T4H-T5H. Results are expressed as medians [minimum; maximum] (Mann-Whitney test, $p < 0.05$, $n = 6$).

Particles T4H-T5H	CEF Infusion Set with Vancomycin	CEF Infusion Set without Vancomycin	P
**≥2 µm**	55,141 [19,641–66,060]	53,449 [36,624–85,663]	>0.9999
**≥5 µm**	9,475 [2,028–16,688]	13,495 [9,819–22,646]	0.0931
**≥10 µm**	1,168 [327–1,837]	1,321 [1,141–2,183]	0.1797
**≥15 µm**	331 [81–516]	528 [348–887]	0.0649
**≥25 µm**	58 [14–64]	74 [27–222]	0.4545
**Total**	86,246 [40,160–100,874]	77,000 [58,118–121,362]	0.8182

Table S5. Number of particles ≥2 µm, ≥5 µm, ≥10 µm, ≥15 µm, ≥25 µm and total on CEF infusion sets with and without filter on the emergency route over the period T0-T8H. Results are expressed as medians [minimum; maximum] (Mann-Whitney test, $p < 0.05$, $n = 6$).

Particles T0-T8H	CEF Infusion Set without Filter	CEF Infusion Set with Filter	P
**≥2 µm**	56,465 [22,352–68,051]	1541 [220–2360]	0.0022
**≥5 µm**	9794 [3087–17,064]	291 [71–657]	0.0022
**≥10 µm**	1238 [338–1884]	22 [8–46]	0.0022
**≥15 µm**	342 [83–526]	8 [1–20]	0.0022
**≥25 µm**	59 [14–64]	3 [0–12]	0.0022
**Total**	89,389 [44,763–104,972]	2254 [322–3,642]	0.0022

Table S6. Number of particles ≥2 µm, ≥5 µm, ≥10 µm, ≥15 µm, ≥25 µm and total on CEF infusion sets with and without filter on the emergency route over the period T4H-T5H. Results are expressed as medians [minimum; maximum] (Mann-Whitney test, $p < 0.05$, $n = 6$).

Particles T4H-T5H	CEF Infusion Set without Filter	CEF Infusion Set with Filter	P
**≥2 µm**	55,141 [19,641–6060]	296 [57–681]	0.0022
**≥5 µm**	9475 [2028–16,688]	44 [31–238]	0.0022
**≥10 µm**	1168 [327–1837]	13 [1–25]	0.0022
**≥15 µm**	331 [81–516]	3 [1–17]	0.0022
**≥25 µm**	58 [14–64]	1 [0–12]	0.0022
**Total**	86,246 [40,160–100,874]	475 [85–988]	0.0022
